# Adoption of a laboratory EMR system and inappropriate laboratory testing in Ontario: a cross-sectional observational study

**DOI:** 10.1186/s12913-021-06296-5

**Published:** 2021-04-06

**Authors:** Nadine Chami, Silvy Mathew, Sharada Weir, James G. Wright, Jasmin Kantarevic

**Affiliations:** 1 Ontario Medical Association, Economics, Policy & Research Department, 150 Bloor St. W, Suite 900, Toronto, ON M5S 3C1 Canada; 2 MyFamilyMD, 396 St. Clair Ave. W, Toronto, ON M5P 3N3 Canada

**Keywords:** EMR, Inappropriate laboratory testing, Primary care models

## Abstract

**Background:**

Electronic medical record (EMR) systems have the potential to facilitate appropriate laboratory testing. We examined three common medical tests in primary care—hemoglobin A1c (HbA1c), lipid, and thyroid stimulating hormone (TSH)— to assess whether adoption of a laboratory EMR system in Ontario had an impact on the rate of inappropriate testing among primary care physicians.

**Methods:**

We used FY2016–17 population-level laboratory data to estimate the association between adoption of a laboratory EMR system and the rate of inappropriate testing. Inappropriate testing was assessed based on recommendations for screening, monitoring, and follow-up that take into account risk factors related to patient age and certain clinical conditions. To overcome the problem of potential endogeneity of physician choice to use the EMR, the EMR penetration rate in the physician’s geographical area of practice was used as an instrumental variable in an ordinary least squares (OLS) regression. We then simulated the change in the rate of inappropriate testing, by physician payment model, as the EMR penetration rate increased from the baseline percentage.

**Results:**

The simulation models showed that an increase in the rate of EMR penetration from a baseline average was associated with a statistically significant decrease in inappropriate hbA1c and lipid testing, but a statistically insignificant increase in inappropriate TSH testing. The impact of EMR penetration also varied by payment model.

**Conclusions:**

This study demonstrated a positive association between availability of an EMR system and appropriate service utilization. Varying impacts of the EMR system availability by primary care payment model may be reflective of different incentives or attributes inherent in payment models. Policies to encourage physicians to increase their use of laboratory EMR systems could improve the quality and continuity of patient care.

**Supplementary Information:**

The online version contains supplementary material available at 10.1186/s12913-021-06296-5.

## Background

Appropriate clinical testing is an important aspect of high-quality medical care [1], and there has been significant interest in reducing potentially unnecessary laboratory and diagnostic testing in recent years [2, 3]. Advances in information technology, such as electronic medical record (EMR) systems, have the potential to facilitate more appropriate laboratory testing and support change in physician test ordering practices by providing physicians with convenient access to patients’ past and current laboratory test results. Studies have found that the use of EMRs had a positive impact on reducing repeat diagnostic testing, where inappropriate testing was defined as a deviation from clinical guidelines or from test-specific intervals [4–8]. In 2014, access to an EMR system with laboratory information, known as the Ontario Laboratories Information System (OLIS), became available to many primary care physicians across Ontario. OLIS is a central repository for all laboratory tests done in Ontario and provides authorized physicians access to a complete and comprehensive history of community, private, and hospital laboratory test orders and results [9]. Benefits of OLIS include progress monitoring of treatments, chronic disease management support, timely access to information that makes it easier for physicians to view current and past test results and to make treatment decisions at the point-of-care, and better care coordination among physicians in different practices and within health care teams [10]. Physicians access patient laboratory reports in OLIS by logging in through the EMR system and then setting a timeline to review previously completed laboratory tests for each specific date. OLIS captures laboratory information of patients moving between hospital, practitioner’s office, home care and long-term care settings, which ensures fewer gaps in patient information. The greater availability and sharing of patient information in OLIS may reduce the number of unnecessary laboratory tests [10]. However, the effect of the adoption of OLIS on inappropriate testing is not well understood.

This study examined the association between widespread availability of a laboratory EMR system and inappropriate testing among primary care physicians. We assessed inappropriate testing based on recommendations for screening, monitoring, and follow-up of each test for patients taking into account pertinent risk factors related to age and certain clinical conditions for three common laboratory tests used in primary care: hemoglobin A1c (HbA1c), lipid, and thyroid stimulating hormone (TSH).

## Methods

We conducted a cross-sectional observational study of the effect of EMR penetration on the rate of inappropriate testing in fiscal year (FY) 2016/17. Data were anonymized and acquired from the Ontario Ministry of Health and Long-Term Care (MOHLTC) under a data-sharing agreement with the Ontario Medical Association (OMA).

### Data sources

Individual community laboratory test-level data were obtained from the Ontario Health Insurance Plan (OHIP) laboratory claims database for FY 2016/17. Data on physician’s age, sex, practice location area, and associated payment model were obtained from the OHIP Corporate Provider Database, and data on patient’s age and sex were obtained from the Registered Persons Database. Information on the percentage of EMR uptake (penetration) in FY 2016/17 by geographical area, known as local health integration network (LHIN) in Ontario, were provided by OntarioMD [11].

Diagnostic information was extracted to summarize patient-level clinical complexity by processing physician billing data from OHIP claims, hospital discharge records from Canadian Institute for Health Information’s (CIHI) Discharge Abstract Database, and in-hospital and community-based ambulatory care records from CIHI’s National Ambulatory Care Reporting System using the CIHI Population Grouping Methodology [12, 13]. CIHI’s Pop Grouper, as it is more commonly known, pools diagnostic code data (10,000 ICD-9 and 18,000 ICD-10 codes) from all available care settings to create a comprehensive set of 226 clinically-meaningful Health Conditions representing chronic and acute illness [13]. Detailed information on the CIHI Pop Grouper is provided in Additional file 1: Appendix A.

### Study population

The study population included all patients eligible and registered for OHIP coverage in FY 2016/17 who had at least one out-of-hospital hbA1c, TSH, or lipid test ordered by a primary care physician. The unit of analysis was the primary care physician. Physicians who ordered less than 100 specific labs per year may not represent the general population of primary care practitioners (e.g., they may be new to practice, employed part-time, acting in temporary roles, or may have primary care as a secondary specialty) and were excluded from the analysis to avoid biasing results.

### Access to laboratory EMR system

Individual physician use of the laboratory EMR may be endogenous since conscientious physicians may be both more likely to use OLIS and to follow guidelines for appropriate test ordering. Therefore, the percentage of EMR uptake (penetration) in FY 2016/17 within a physician’s geographical area was used as an instrumental variable in an ordinary least squares (OLS) regression. A table and map listing the EMR penetration rate by geographical area (LHIN) can be found in Additional file 1: Appendix B1 and B2.

### Inappropriate laboratory testing

The laboratory tests analyzed in this study were hbA1c, lipid, and TSH. These tests were chosen because they are all commonly ordered tests for screening, chronic illness assessment or monitoring by primary care physicians. We employed two different approaches to define inappropriate testing. Firstly, we used repeat testing as a measure of inappropriateness based on accepted guidelines for screening, monitoring, and follow-up of each test that treats all patients the same, regardless of age and clinical history and is in line with prior research on inappropriate testing of hbA1c, lipid, and TSH [14, 15]. Using this definition, any hbA1c test done within 3 months of a previous test (plus/minus 2 weeks), a TSH test done within 8 weeks of a previous test (plus/minus 2 weeks), and a lipid test done within 3 months of a previous test (plus/minus 2 weeks) were deemed to have been ordered inappropriately [14–19].

We then introduced an enhanced approach to measure inappropriateness wherein criteria for hbA1c, TSH, and lipid testing were derived from guidelines and recommendations on testing for patients with risk factors based on age and/or clinical conditions that justified ordering a laboratory test [16–21]. In the enhanced framework, hbA1c tests were considered inappropriate as follows: tests done within 3 months of a previous test for patients without a specific clinical condition in the past year, tests done on patients younger than 40 without a specific clinical condition such as diabetes, and tests done more than once per year on patients aged 40 and older without a specific clinical condition [18]. For TSH testing, inappropriate testing included any test done for patients of any age without a specific clinical condition, and any test done within 3 months of a previous test for patients without a specific clinical condition in the past year [19, 20]. For lipid testing, any test done for patients younger than 40 without a specific clinical condition such as hypertension, any test done within 3 years of a previous test for patients 40 and older without a specific clinical condition, and any test done within 3 months of a previous test on patients without a specific clinical condition were considered inappropriate [21]. This approach to measuring inappropriateness emphasized both specificity, which limits the number of false positives (tests that were defined as inappropriate when in fact they were appropriate), and sensitivity, which limits the number of false negatives (tests that were defined as appropriate when in fact they were inappropriate).

Clinical conditions were identified in the data using patient-level diagnostic information from the CIHI Pop Grouper. The guidelines for each test based on patient age and clinical diagnosis, as well as a list of the specific clinical conditions/concerns where ordering additional laboratory tests may be warranted to guide clinical course, can be found in Additional file 1: Appendix C.

### Patient clinical complexity

Patient risk scores (which reflect patient complexity as proxied by cost risk) were calculated based on the presence of one or more CIHI Health Conditions in the previous five years. These scores were then normalized to the population of patients included in this study who had laboratory tests done in Ontario in FY 2016/17 so that patients with a risk score greater than 1 were more complex than average for the study population, while those with a risk score less than 1 were less complex than average.

### Physician choice of payment model

We controlled for the physicians’ choice of payment and practice models: traditional fee-for-service (FFS); a retrospective payment model with elements of pay-for-performance (enhanced FFS); a predominantly prospective payment model mixed with FFS and elements of pay-for-performance (blended capitation); and a blended capitation model with an interdisciplinary team of family physicians, nurse practitioners, registered nurses, social workers, dietitians, and other health care professionals (interdisciplinary blended capitation model). Primary care physicians in other payment models were excluded, since these models comprise only a small proportion of the province’s physicians.

### Statistical analysis

First, we compared basic physician and patient descriptive statistics for the four different payment models. The descriptive statistics were presented separately for hbA1c, lipid, and TSH tests, and included the total number of physicians and patients, average patient age, percentage of male patients, average patient complexity, average normalized patient complexity, average physician age, average percentage of male physicians, average percentage of EMR uptake geographically, average percentage of inappropriate laboratory tests ordered, and average percentage of patients with at least one inappropriate test by payment model. The total number of patients in each payment model was not mutually exclusive. If the same patient received an hbA1c test order from both an FFS physician and a blended capitation physician, that patient would be counted twice in this metric. Descriptive statistics on both the standard and enhanced measures were reported.

Second, OLS regression was used to estimate the association between adoption of a laboratory EMR system and the rate of inappropriate testing for hbA1c, lipid, and TSH laboratory tests. Here, the enhanced measure of inappropriateness, based on patient age and clinical conditions, were employed. Physician and patient characteristics including physician age, physician sex, average patient age, percentage of male patients, and average patient complexity were included as control variables in the regression. The OLS model used for calculating the overall impact of EMR penetration on inappropriate testing was as follows:

1 $$ Y={a}_0+{b}_1X+{b}_2Z $$

where *Y* is the rate of inappropriate testing at the physician-level, *X* is the set of physician and patient control variables mentioned above, and *Z* is the percentage of penetration of the laboratory EMR system by geographical area. A second model was used to estimate the impact of EMR penetration on inappropriate testing by physician payment model as follows:

2 $$ Y={a}_0+{b}_1X+{b}_2Z+{c}_1 EFFS+{c}_2 BC+{c}_3 IBC+{d}_1\left( EFFS\ast Z\right)+{d}_2\left( BC\ast Z\right)+{d}_3\left( IBC\ast Z\right) $$

where *EFFS* is a dummy variable equal to 1 if the physician was in an enhanced FFS model and 0 otherwise; *BC* is a dummy variable equal to 1 if the physician was in a blended capitation model and 0 otherwise; *IBC* is a dummy variable equal to 1 if the physician was in an interdisciplinary blended capitation model and 0 otherwise. These dummy variables for physician payment model were included to estimate the association between adoption of the EMR system and the rate of inappropriate testing separately for each payment model. The reference, or omitted, category was the FFS model. The interaction variables of EMR penetration with the physician’s payment model represented the marginal association between the percentage of inappropriate testing and EMR penetration of FFS physicians compared to other physicians (enhanced FFS, blended capitation and interdisciplinary blended capitation).

Using the OLS regression results from the two models above, we simulated the change in the rate of inappropriate testing as the EMR penetration rate increased from the baseline percentage for the average rate of EMR penetration (*Z* = EMR penetration mean at baseline). Results were reported in increments from increases of 5 percentage points up to 25 percentage points. For each physician payment model, the simulation would be calculated using model (2) as

3 $$ {Y}_{FFS}={a}_0+{b}_1X+{b}_2Z $$

4 $$ {Y}_{EFFS}={a}_0+{b}_1X+{b}_2Z+{c}_1 EFFS+{d}_1\left( EFFS\ast Z\right) $$

5 $$ {Y}_{BC}={a}_0+{b}_1X+{b}_2Z+{c}_2 BC+{d}_2\left( BC\ast Z\right) $$

6 $$ {Y}_{IBC}={a}_0+{b}_1X+{b}_2Z+{c}_3 IBC+{d}_3\left( IBC\ast Z\right) $$

where the coefficients from the OLS regression were multiplied by the descriptive statistic averages, and *Z* is the EMR penetration rate starting at baseline and increased by 5 percentage points for each simulation. The baseline value was the average EMR penetration rate for physicians in the sample for each laboratory test. All analyses were conducted using Stata 15 (College Station, TX).

## Results

### Descriptive statistics

Table 1 shows the descriptive statistics for ordering of hbA1c, lipid, and TSH tests by payment model for FY 2016/17. Physicians in the interdisciplinary blended capitation model had the lowest rate of inappropriate testing for hbA1c tests, while EFFS physicians had the lowest rate for inappropriate lipid and TSH tests compared to other models. The interdisciplinary blended capitation model also had the highest normalized patient complexity and percentage of EMR penetration rate for all three laboratory tests.

**Table 1:** Descriptive statistics on hbA1c, TSH and lipid tests in FY2016/17 by payment model

Payment Model	FFS	EFFS	BC	IBC	Total
**HbA1c Tests**					
Total number of physicians	1768	2139	2424	1960	8291
Total number of patients	473,345	1,041,662	962,224	643,576	3,064,417
Average physician age	47.0	53.3	52.0	49.4	50.7
Average % of male physicians	53.5%	57.2%	54.1%	52.2%	54.3%
Average patient age	56.3	56.0	59.7	60.9	58.3
Average % of male patients	45.5%	45.4%	45.6%	46.9%	45.9%
Average patient complexity	3.93	3.44	3.68	4.05	3.76
Average normalized patient complexity	1.03	0.90	1.01	1.11	1.00
% EMR penetration	34.9%	35.0%	34.4%	37.0%	35.3%
Average % of inappropriate lab tests	5.4%	4.4%	4.3%	4.6%	4.6%
Average % of inappropriate lab tests based on patient age and clinical condition	6.0%	4.6%	4.2%	3.9%	4.6%
Average % of patients with at least one inappropriate lab test	9.5%	7.6%	8.4%	9.1%	8.4%
Average % of patients with at least one inappropriate lab test based on patient age and clinical condition	7.5%	6.2%	5.7%	5.3%	6%
**TSH Tests**					
Total # of physicians	1807	2196	2459	1917	8379
Total # of patients	511,432	1,164,682	1,003,941	584,782	3,198,964
Average physician age	50.1	53.5	53.5	50.9	52.5
Average % of male physicians	57.0%	59.9%	58.3%	56.7%	58.4%
Average patient age	51.6	50.9	55.1	56.1	53.2
Average % of male patients	36.0%	37.6%	35.5%	33.4%	36.0%
Average patient complexity	3.53	3.12	3.44	3.85	3.41
Average normalized patient complexity	1.03	0.91	1.01	1.13	1.00
% EMR penetration	34.9%	35.0%	34.4%	37.0%	35.3%
Average % of inappropriate lab tests	4.1%	2.9%	2.9%	3.4%	3.3%
Average % of inappropriate lab tests based on patient age and clinical condition	5.1%	3.3%	4.1%	4.9%	4.3%
Average % of patients with at least one inappropriate lab test	4.9%	3.2%	3.1%	3.4%	3.5%
Average % of patients with at least one inappropriate lab test based on patient age and clinical condition	5.6%	3.7%	4.1%	4.7%	4.3%
**Lipid Tests**					
Total # of physicians	1562	2153	2422	1876	8013
Total # of patients	461,066	1,146,751	1,005,537	594,909	3,161,228
Average physician age	50.8	54.3	54.0	51.0	53.1
Average % of male physicians	61.7%	63.9%	63.1%	61.8%	63.0%
Average patient age	54.5	54.5	58.8	60.0	56.8
Average % of male patients	46.8%	47.0%	47.9%	49.2%	47.6%
Average patient complexity	3.08	2.90	3.13	3.37	3.08
Average normalized patient complexity	1.00	0.94	1.01	1.09	1.00
% EMR penetration	34.9%	35.0%	34.4%	37.0%	35.3%
Average % of inappropriate lab tests	4.8%	3.3%	2.8%	2.8%	3.3%
Average % of inappropriate lab tests based on patient age and clinical condition	2.1%	1.5%	1.5%	1.5%	1.6%
Average % of patients with at least one inappropriate lab test	5.8%	4.0%	3.5%	3.3%	4.0%
Average % of patients with at least one inappropriate lab test based on patient age and clinical condition	2.7%	1.9%	1.8%	1.8%	1.9%

### OLS regression simulation models

**Fig. 1:**
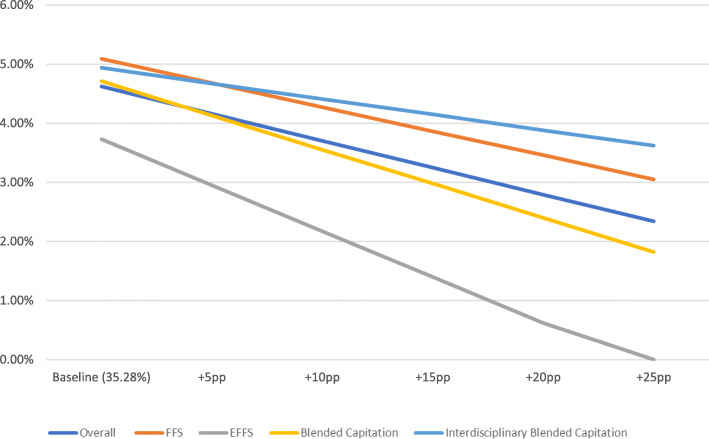
Inappropriate hbA1c testing rate plus simulated effect of increasing EMR penetration rate by selected percentage points (baseline+Xpp)

**Fig. 2:**
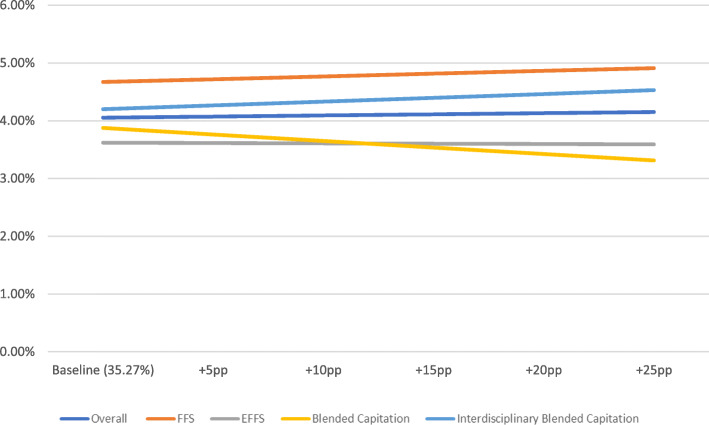
Inappropriate TSH testing rate plus simulated effect of increasing EMR penetration rate by selected percentage points (baseline+Xpp)

**Fig. 3:**
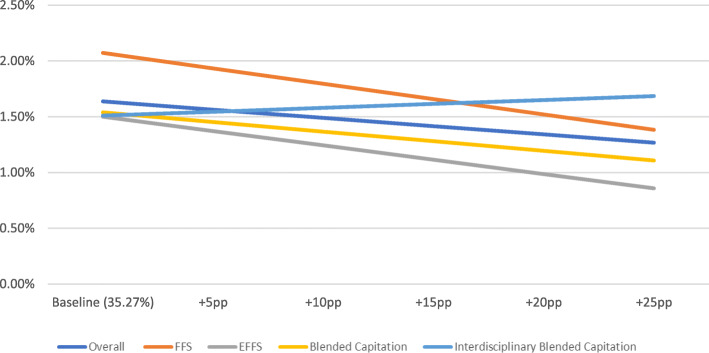
Inappropriate lipid testing rate plus simulated effect of increasing EMR penetration rate by selected percentage points (baseline+Xpp)

The OLS regression results are presented in Table 2 (overall results) and Table 3 (results by payment model). Figs. 1, 2 and 3 present the results of the simulation models using these OLS regressions. Rates shown were adjusted using OLS regression to control for physician age and sex as well as average patient age, sex, and complexity among patients who were tested by the physician. If the simulated rate of inappropriate testing was negative, the rate was presented as 0% or no effect. The simulation models showed that at the baseline EMR penetration rate of 35.3%, the rates of inappropriate hbA1c, TSH, and lipid testing were 4.6, 4, and 1.6%, respectively. An increase in the EMR penetration rate of 25 percentage points from baseline was associated with a statistically significant decrease in inappropriate hbA1c testing to 2.3%, a statistically insignificant increase in inappropriate TSH testing to 4.2% and a statistically significant decrease in inappropriate lipid testing to 1.3% (Figs. 1, 2 and 3).

**Table 2:** OLS results (overall); outcome variable = % inappropriate laboratory tests

Variable	HbA1c	TSH	Lipid
**%** OLIS Penetration	−0.0913^a^	0.0049	−0.0148^a^
**Control Variables**			
Average patient age	−0.0039^a^	0.0003^a^	−0.0003^a^
Average percentage of male patients	−0.0297^a^	−0.0331^a^	−0.0043
Average patient complexity	0.0036^a^	0.0089^a^	0.0048^a^
Sex of physician (=1 if male; =0 if female)	0.0050^a^	−0.0015	0.0004
Physician age	−0.0002^a^	−0.0004^a^	0.0000^a^
Constant	−0.0039^a^	0.0229^a^	0.0242^a^
Number of physicians	8291	8379	8013
R^2^	0.492	0.285	0.099

^a^significant at 1% level

**Table 3:** OLS results by payment model; outcome variable = % inappropriate laboratory tests

Variable	HbA1c	TSH	Lipid
**%** OLIS Penetration	−0.0818^a^	0.0097	−0.0276^a^
**Payment model**: reference category: FFS			
EFFS	0.0122	−0.0067	−0.0064
BC	0.0082	0.0034	−0.0089^c^
IBC	−0.0118	−0.0059	−0.0178^a^
**Payment model x % OLIS Penetration:** reference category: FFS x **%** OLIS Penetration			
EFFS x **%** OLIS Penetration	−0.0734^a^	−0.0109	0.0019
BC x **%** OLIS Penetration	−0.0340	−0.0322	0.0103
IBC x **%** OLIS Penetration	0.0291	0.0034	0.0346^a^
**Control Variables**			
Average patient age	−0.0041^a^	0.0004^a^	−0.0003^a^
Average percentage of male patients	−0.0301^a^	−0.0330^a^	−0.0044
Average patient complexity	0.0035^a^	0.0085^a^	0.0045^a^
Sex of physician (=1 if male; =0 if female)	0.0053^a^	−0.0016^c^	0.0002
Physician age	−0.0001^b^	−0.0003^a^	0.0001^a^
Constant	0.3201^a^	0.0242^a^	0.0290^a^
Number of physicians	8291	8379	8013
R^2^	0.513	0.298	0.118

^a^significant at 1% level

^b^significant at 5% level

^c^significant at 10% level

Results by payment model indicated that the rate of inappropriate testing for the three laboratory tests at the baseline EMR penetration rate was highest for FFS physicians. Fig.1 shows that an increase in the EMR penetration rate of 25 percentage points from baseline would be associated with a decrease in inappropriate hbA1c testing for all payment models. On the other hand, an increase in EMR penetration rate was associated with a slight increase in the rate of inappropriate TSH testing (Fig. 2), but the OLS coefficients were statistically insignificant. For lipid testing, an increase in the EMR penetration rate of 25 percentage points from baseline would result in a decrease in inappropriate testing, except for in the case of the interdisciplinary blended capitation model where there was a slight increase (Fig. 3).

## Discussion

This study demonstrated an association between the adoption of a widespread laboratory EMR system and a reduction in the rate of inappropriate hbA1c and lipid tests. EMR penetration, in contrast, was not related to inappropriate TSH testing. This finding is in line with results from previous studies that showed evidence of overutilization of TSH testing in Ontario family medicine practices [22] and no reduction in TSH testing upon introduction of an intervention aimed at reducing TSH laboratory service utilization in Ontario [23]. As discussed in these studies, interventions targeting reduction in TSH testing may not be effective due to inconsistent screening guidelines and clinician uncertainty in assessing specific symptoms related to the thyroid. Unlike hbA1c and lipid tests which are discrete tests to assess for and guide around specific conditions, TSH testing is used beyond accepted guidelines to help clarify uncertain clinical pictures. This is because the thyroid hormones physiologically affect the body in innumerous ways, both directly and indirectly. Therefore, diagnostic pathways unrelated to accepted guidelines will often suggest TSH testing when there is clinical uncertainty. Although one of the goals of OLIS is to reduce repeat testing, due to clinical ambiguity of certain tests such as TSH, higher uptake of the laboratory EMR may not necessarily be associated with reductions in inappropriate use for all tests.

This study also showed varying impacts of the EMR system by primary care payment model, which may reflect different incentives or attributes inherent in payment models to appropriately order tests. For example, switching from an enhanced FFS model to a blended capitation or an interdisciplinary blended capitation model was shown to be associated with a 3 and 9% reduction, respectively, in inappropriate hbA1c testing, suggesting an improvement in continuity of care for blended capitation physicians [24]. Self-selection into a payment/practice model may have been related to physician preference regarding practice style [25], which may also have affected the choice of accessing OLIS prior to ordering a laboratory test where physicians need to individually review previously completed tests. This may be regarded as a time constraint for some physicians, especially those in retrospective payment models such as FFS. Since selection into the payment model may be correlated with selection of adopting the laboratory EMR system, and we did not control for this selection, the impact of EMR adoption on inappropriate testing should be interpreted as an association rather than a causal relationship.

Prior studies of inappropriate utilization have used deviation from guidelines that do not take into account patient age or clinical condition criteria to define inappropriate repeat testing [5, 14, 15]. However, guidelines, as the name suggests, are not hard and fast rules and there are often general and sensible clinical reasons to deviate from them. For example, an adolescent who is prescribed oral isotretinoin to treat severe acne will have monthly lipid tests well before the recommended screening age guideline of 40 years old. In this study we constructed a measure of inappropriateness that used patient age and clinical criteria to identify laboratory tests that may have been requested earlier than the recommended guidelines but should not be considered medically inappropriate or unnecessary.

To our knowledge, this is the first study that attempted to measure the effect of the adoption of the laboratory EMR system on rates of inappropriate testing in Ontario. Owing to the universal public funding of the Ontario healthcare system, we were able to capture nearly all out-of-hospital laboratory tests ordered by the physicians in our study sample.

OLIS provides easier access to laboratory testing data and information for health care providers, but it does not provide them with laboratory guidelines and recommendations. If soft nudges were imbedded in the EMR system, this might be effective at encouraging desirable physician test ordering behavior. For example, an alert system that notifies the physician when a test is being ordered before the recommended time interval for repeat testing may reduce inappropriate testing. Other policies to promote greater adoption of OLIS among primary care physicians include strategies or technologies that allow for easier access of OLIS within an EMR, removing time constraints and disincentives of use, such as allowing physicians to search for specific completed laboratory tests without manually navigating through each patient laboratory report.

### Limitations

There were several potential limitations to our study. First, tests ordered by primary care physicians outside a primary care practice setting were not available in our data. However, this limitation did not likely affect our results because the tests included in this study were selected for their outpatient-focused qualities and are rarely ordered in acute care settings. Second, we lacked data on other clinical risk factors, such as family history and ethnicity, that may have a role in the decision-making related to test ordering. These factors are not captured in the CIHI Pop Grouper directly. Third, due to the cross-sectional nature of the study, we could not infer causality of EMR adoption on rates of inappropriate testing. Lastly, the exact mechanism by which EMR adoption affects appropriate use of testing remains an area for future research.

## Conclusion

This study demonstrated a positive association between availability of a laboratory EMR system and appropriate service utilization. Policies to encourage or incentivize physicians to increase their use of EMR systems could not only lower unnecessary health care costs but also improve the quality and continuity of patient care. As health care provision becomes more complex in response to aging populations and advances in technology, it is imperative that healthcare systems determine ways to promote comprehensiveness and continuity.

## Supplementary Information

**Additional file 1: Appendix A.** Overview of CIHI’s Population Grouping Methodology. **Appendix B1.** Map of percentage of OLIS uptake by physicians per LHIN. **Appendix B2.** Table of percentage of OLIS uptake by physicians per LHIN. **Appendix C.** Guidelines developed to identify inappropriate (too frequent or redundant) tests.

## Data Availability

The datasets generated and analyzed during the current study are not publicly available due to confidential physician and patient data.

## Abbreviations

EMR: Electronic medical record
HbA1c: Hemoglobin A1c
TSH: Thyroid stimulating hormone
OLIS: Ontario Laboratories Information System
FY: Fiscal year
MOHLTC: Ontario Ministry of Health and Long-Term Care
OMA: Ontario Medical Association
OHIP: Ontario Health Insurance Plan
LHIN: Local health integration network
CIHI: Canadian Institute for Health Information
OLS: Ordinary least squares
FFS: Fee-for-service
EFFS: Enhanced fee-for-service
BC: Blended capitation
IBC: Interdisciplinary blended capitation

## References

[1] Wilson ML (2002). Appropriate use of clinical microbiology tests. Clin Lab Med.

[2] Wendy Levinson. The Canadian Public Needs to Know More is Not Always Better When it Comes to Healthcare [Internet]. Choosing Wisely Canada; 2016. Available from: https://choosingwiselycanada.org/perspective/more-is-not-always-better-healthcare/. Accessed 20 Feb 2020.

[3] Canadian Institute for Health Information (2017). Unnecessary care in Canada: technical report.

[4] Canada Health Infoway. The emerging benefits of electronic medical record use in community-based care 2013. https://infoway-inforoute.ca/en/component/edocman/1224-the-emerging-benefits-of-electronic-medical-record-use-in-community-based-care-full-report/view-document?Itemid=101/ (Accessed 20 Feb 2020).

[5] Georgiou A, Vecellio E, Li L (2015). The impact of an electronic medical record on repeat laboratory test ordering across four Australian hospitals. Stud Health Technol Inform.

[6] Tierney WM, McDonald CJ, Martin DK (1987). Computerized display of past test results. Ann Intern Med.

[7] Krasowski MD, Chudzik D, Dolezal A, Steussy B, Gailey MP, Koch B, Kilborn SB, Darbro BW, Rysgaard CD, Klesney-Tait JA (2015). Promoting improved utilization of laboratory testing through changes in an electronic medical record: experience at an academic medical center. BMC Med Inform Decision Making.

[8] Bates DW, Kuperman GJ, Rittenberg E, et al. A randomized trial of a computer-based intervention to reduce utilization of redundant laboratory tests. Am J Med. 1999:106144–50. 10.1016/s0002-9343(98)00410-0 10230742

[9] Lab Results [Internet]. eHealth Ontario. Available from: https://ehealthontario.on.ca/en/health-care-professionals/lab-results. Accessed 15 Apr 2020.

[10] eHealth Ontario. Ontario Laboratories Information System. eHealth Ontario; 2012. Available from: https://collections.ola.org/mon/26010/319773.pdf. Accessed 13 July 2020.

[11] OntarioMD. LHINs Q4 Report January–March 2017 2017. https://www.ontariomd.ca/documents/lhin%20reports/lhin%20report%20for%20q4%202016-17.pdf (Accessed 20 Feb 2020).

[12] Canadian Institute for Health Information. Population Grouping Methodology. 2017 https://www.cihi.ca/en/document/population-grouping-methodology-0/ (Accessed Feb 2020).

[13] Li Y, Weir S, Steffler M, Shaikh S, Wright JG, Kantarevic J (2019). Using diagnoses to estimate health care cost risk in Canada. Med Care.

[14] Morgen EK, Naugler C (2015). Inappropriate repeats of six common tests in a Canadian city: a population cohort study within a laboratory informatics framework. Am J Clin Pathol.

[15] Chami N, Simons JE, Sweetman A (2016). Rates of inappropriate laboratory test utilization in Ontario. Clin Biochem.

[16] Berard LD, Siemens R, Woo V (2018). Diabetes Canada 2018 clinical practice guidelines for the prevention and Management of Diabetes in Canada: monitoring Glycemic control. Can J Diabetes.

[17] American Thyroid Association. Q and A: TSH (thyroid stimulating hormone) [Internet]. Alexandria, VA: American Thyroid Association. Available from: https://www.thyroid.org/patient-thyroid-information/what-are-thyroid-problems/q-and-a-tsh-thyroid-stimulating-hormone/#:~:text=A%20TSH%20blood%20test%20should,have%20reached%20a%20steady%20state. Accessed 7 Jan 2021.

[18] Diabetes Canada. 2018 Clinical Practice Guidelines: Quick Reference Guide. Diabetes Canada; 2018. Available from: http://guidelines.diabetes.ca/docs/CPG-quick-reference-guide-web-EN.pdf. Accessed 20 Feb 2020.

[19] Guidelines & Protocols Advisory Committee. Thyroid Function Testing in the Diagnosis and Monitoring of Thyroid Function Disorder. British Columbia Ministry of Health; 2018. Available from: https://www2.gov.bc.ca/assets/gov/health/practitioner-pro/bc-guidelines/thyroid-function-testing.pdf. Accessed 20 Feb 2020.

[20] Toward Optimized Practice (TOP) Endocrine Working Group. Investigation and management of primary thyroid dysfunction clinical practice guideline. Edmonton: Toward Optimized Practice; 2014. Available from: http://www.topalbertadoctors.org. Accessed Feb 2020.

[21] Tobe SW, Stone JA, Anderson T, Bacon S, Cheng AYY, Daskalopoulou SS, Ezekowitz JA, Gregoire JC, Gubitz G, Jain R, Keshavjee K, Lindsay P, L’Abbe M, Lau DCW, Leiter LA, O’Meara E, Pearson GJ, Rabi DM, Sherifali D, Selby P, Tu JV, Wharton S, Walker KM, Hua-Stewart D, Liu PP (2018). Canadian cardiovascular harmonized National Guidelines Endeavour (C-CHANGE) guideline for the prevention and management of cardiovascular disease in primary care: 2018 update. CMAJ.

[22] Birk-Urovitz E, Del Giudice EM, Meaney C (2017). Use of thyroid-stimulating hormone tests for identifying primary hypothyroidism in family medicine patients. Can Fam Physician.

[23] Chami N, Li Y, Weir S, Wright JG, Kantarevic J (2021). Effect of strict and soft policy interventions on laboratory diagnostic testing in Ontario, Canada: a Bayesian structural time series analysis. Health Policy.

[24] Chami N, Sweetman A (2019). Payment models in primary health care: a driver of the quantity and quality of medical laboratory utilization. Health Econ.

[25] Rudoler DM (2015). Paying for primary care: The relationship between payment change and primary care physician behaviour.

