# Supplementary material for: Adoption of a laboratory EMR system and inappropriate laboratory testing in Ontario: a cross-sectional observational study
Source: BMC Health Serv Res. 2021 Apr 6;21:307. doi: 10.1186/s12913-021-06296-5 (PMC8025377; doi:10.1186/s12913-021-06296-5)
Supplement: Supplementary file 1 — Additional file 1: Appendix A. Overview of CIHI’s Population Grouping Methodology. Appendix B1. Map of percentage of OLIS uptake by physicians per LHIN. Appendix B2. Table of percentage of OLIS uptake by physicians per LHIN. Appendix C. Guidelines developed to identify inappropriate (too frequent or redundant) tests. [file 12913_2021_6296_MOESM1_ESM.docx]

**Appendix: Adoption of a Laboratory EMR System and Inappropriate Laboratory Testing in Ontario: A Cross-Sectional Observational Study**

## Appendix A: Overview of CIHI’s Population Grouping Methodology

CIHI’s Population Grouping Methodology (also known as CIHI’s Pop Grouper) is a Canadian tool for summarizing patient complexity and predicting health risk using patient-level healthcare administrative data. It was developed using pooled data from three provinces in Canada – Alberta, British Columbia and Ontario – for fiscal years 2010 and 2011 (concurrent period) and fiscal year 2012 (prospective period).

Patient clinical profiles are created by summarizing diagnosis codes recorded in claims during the lookback period. CIHI’s diagnosis grouper uses 10,000 ICD-9 and 18,000 ICD-10-CA diagnosis codes recorded from inpatient stays, day surgeries/procedures, emergency department visits, and physician encounters, as well as DSM-IV codes (from all available settings) and some elements from RAI-MDS 2.0© and RAI-MH© (recorded in the long term care setting) and classifies the diagnosis codes into clinically similar groups of 226 Health Conditions (HCs). HCs include chronic and acute illnesses, disabilities, medical emergencies, signs and symptoms, and other health states, such as pregnancy. Each diagnosis code is linked to a single HC based on algorithms created in consultation with physician experts. Patients may have multiple HCs, but “clinical override rules” mean that the model only counts relatively more serious and non-redundant conditions. For example, a patient with HCs for seizure and epilepsy will have a record of only epilepsy after applying override rules.

The cost of health system users with at least one HC were modelled in an ordinary least squares regression based on the 226 HCs and 460 HC interaction terms. Costs of health system users with no conditions and of health system non-users were based on age and gender only. Predictive risk scores are then generated from the coefficients or model weights which allow the comparison of patient costs relative to others in the study sample or population.

The CIHI Pop Grouper has been shown to outperform the Johns Hopkins ACG® model for a Canadian population^1^. The ability of the Grouper to explain nearly half of the variance in the dependent variable using age, gender, health conditions and condition interactions in the concurrent period (R^2^=0.475) and almost one-tenth in the prospective period (R^2^=0.094) was documented by CIHI using a model validation sample of the three provinces (Alberta, British Columbia and Ontario)^2^ and externally validated for the province of Ontario^3^.

1. Cheng S, Austin P, Wodchis W, et al. Evaluation of Population Groupers. ICES Report, September 2016.
2. Canadian Institute for Health Information. CIHI’s Population Grouping Methodology 1.1 (compiled code): Methodology Report. CIHI, April 2017.
3. Li Y, Weir S, Steffler M, Shaikh S, Wright J, Kantarevic, J. Using Diagnoses to Estimate Healthcare Cost Risk in Canada. Philadelphia, PA: Medical Care. 2019 Nov;57(11):875-881.

**Appendix B1: Map of percentage of OLIS uptake by physicians per LHIN**

**
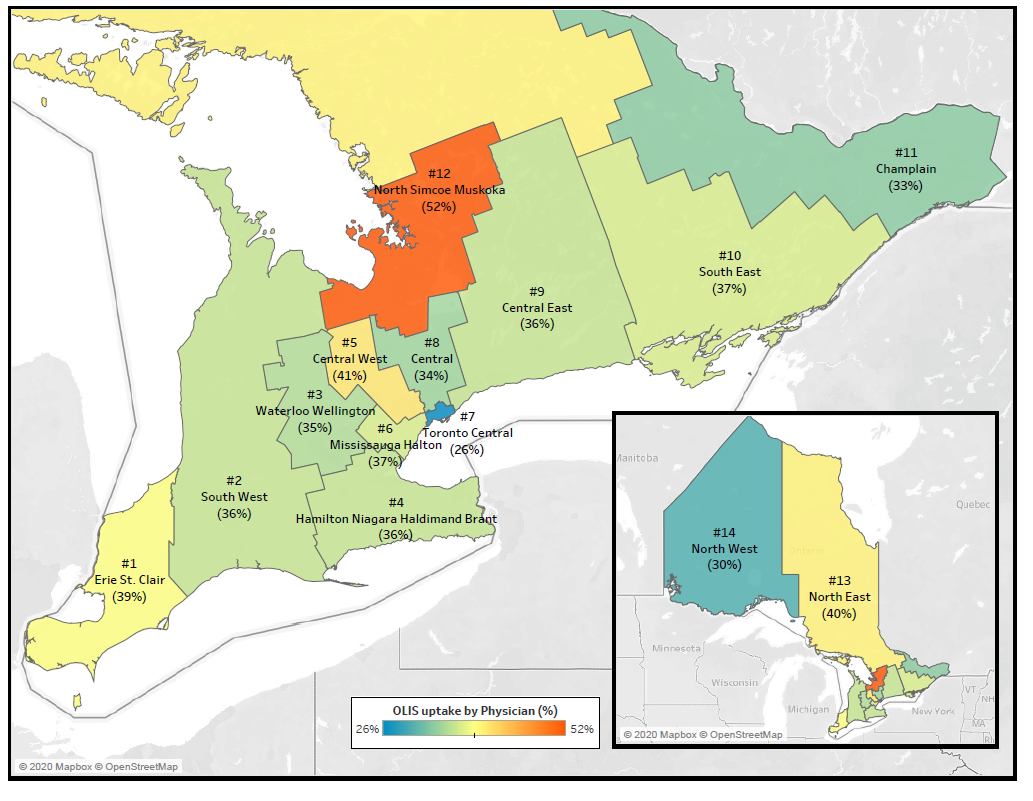
**

Map provided by Statistics Canada. 2018. Health Region Boundary File. Statistics Canada Catalogue no. 82-402-X. Dec 14.
<https://www150.statcan.gc.ca/n1/pub/82-402-x/2018001/hrbf-flrs-eng.htm> (accessed March 10, 2020).

**Appendix B2: Table of percentage of OLIS uptake by physicians per LHIN**

| **LHIN #** | **LHIN Name** | **OLIS penetration rate** |
| --- | --- | --- |
| 1 | Erie St. Clair | 39% |
| 2 | South West | 36% |
| 3 | Waterloo Wellington | 35% |
| 4 | Hamilton Niagara | 36% |
| 5 | Central West | 41% |
| 6 | Mississauga Halton | 37% |
| 7 | Toronto Central | 26% |
| 8 | Central | 34% |
| 9 | Central East | 36% |
| 10 | South East | 37% |
| 11 | Champlain | 33% |
| 12 | North Simcoe Muskoka | 52% |
| 13 | North East | 40% |
| 14 | North West | 30% |

OntarioMD. LHINs Q4 Report January-March 2017. <https://www.ontariomd.ca/documents/lhin%20reports/lhin%20report%20for%20q4%202016-17.pdf>

**Appendix C: Guidelines developed to identify inappropriate (too frequent or redundant) tests**

CIHI groups: “Population Grouping Methodology | CIHI,” 2017

1. **HbA1c guideline**: “Diabetes Canada | Clinical Practice Guidelines—CPG Quick Reference Guide,” n.d.,
2. **any test done age < 40 without the following diagnoses (exclusion criteria):**

**the conditions noted overlap in ICD 9/10 diagnostic codes and the CIHI groups are even broader. Therefore, every condition considered may not be listed by name, but are encompassed in the appropriate CIHI group.*

| Condition | CIHI group |
| --- | --- |
| *Diabetes:*  250 – diabetes  248 – diabetes with ocular complications  790 – other to include IFG/IGT/pre-DM  799 – failure to thrive/other | J12, J02, G03, J06, J07, J08, J09, S81 |
| 650 - pregnancy | M41, M43, M42, N42, N43, N41 |
| *Thyroid:*  240 – simple thyroid goiter  242 – hyperthyroidism  244 - hypothyroidism  245 – thyroiditis | J03, J04, J05 |
| 580 - nephropathy | K01, K41, K81 |
| 350 - neuropathy | A08, A11, A48, A10, A47, A81, A82 |
| 379 - retinopathy | B02, B05, B07, B41 |
| 290 - dementia | A84, Q01, Q81 |
| 278 – obesity  796 – weight loss  787 – eating disorder | J10, J13, Q08 |
| *Rheumatologic:*  696 – psoriasis  714 – RA  710 – lupus  135 –sarcoid  555 – Crohn’s  564 – colitis | F06, H01, H07, H09, I01, I02, I03, I05 |
| *Respiratory:*  493 – asthma  277 – CF  492 – emphysema  496 - COPD | D06, J01, D03, D05 |
| 038, 590 - sepsis | P41, S47 |
| *Psychiatric:*  296 – Bipolar Disorder  295 – Schizophrenia  298 - Psychosis, other  787 – eating disorder | Q02, Q05 |
| 305 - smoking | Q027 |
| 157 – pancreatic cancer | R05F |

1. **If person is aged >=40 years, any HbA1c more frequent than annual +/- four weeks without exclusion criteria**
   1. Exclusion criteria: as above *except for Diabetes group diagnoses*
2. **HbA1c testing more frequently than every three months +/- two weeks at any age without the following diagnoses in the past year** (medications or clinical condition changes may indicate more frequent testing):

| Condition | CIHI group |
| --- | --- |
| 650 - pregnancy | M41, M43, M42, N42, N43, N41 |
| 290 – dementia/delirium | Q81 |
| *Rheumatologic:*  696 – psoriasis  714 – RA  710 – lupus  135 –sarcoid  555 – Crohn’s  564 – colitis | F06, H01, H07, H09, I01, I02, I03, I05 |
| *Respiratory:*  493 – asthma  277 – CF  492 – emphysema  496 - COPD | D06, J01, D03, D05 |
| 038, 590 - sepsis | P41, S47 |
| 157 – pancreatic cancer | R05F |

1. **TSH** guidelines:
2. Alberta “toward optimal practice” guidelines (*Thyroid_guideline.pdf*, n.d.)
3. British Colombia guidelines: (Health, n.d.)
4. **Any screening TSH completed except if the following diagnoses apply:**

**the conditions noted overlap in ICD 9/10 diagnostic codes and the CIHI groups are even broader. Therefore, every condition considered may not be listed by name, but are encompassed in the appropriate CIHI group.*

| Conditions | CIHI groups associated |
| --- | --- |
| *Endocrine:*  193 – malignant neoplasm-thyroid  226 – benign neoplasm-thyroid  240 – simple thyroid goiter  242 – hyperthyroidism  244 - hypothyroidism  245 – thyroiditis  252 - parathyroid gland disorders  250 – diabetes  278 - obesity | B04, B05, B06, B07, J02, J12, J10, J03, J04, J05, J06, J07, J08, R10j |
| *Pregnancy:*  640 - threatened abortion, haemorrhage in early pregnancy  641 - abruptio placentae, placenta praevia  642- pre-eclampsia, eclampsia, toxaemia  643 - vomiting, hyperemesis gravidarum  644 - false labour, threatened labour  645 - prolonged pregnancy (post dates/post maturity pregnancy)  646 - other complications of pregnancy  651 - multiple pregnancy  650 – pregnancy  633 – ectopic | M41, M42, M43, N41, N42, N43 |
| *Menstrual disorder*  626 - amenorrhea, anovulation | L02, L03, L04 |
| *Nervous System:*  191 - malignant neoplasms – brain  345 – epilepsy  780 – headache other, vertigo  340 – MS  350– neuritis  356 - neuralgia | A04, A08, A11, A47, A05, A81 A83, C08,R01A, R02C, R17, R18, J07 |
| *Fertility:*  606 - male infertility  628 – female infertility  895 – family planning | L01, K06 |
| *Psychiatric:*  296 – Bipolar Disorder  295 – Schizophrenia  300 – Depression/Anxiety  301 – OCD/Personality Disorder  311 – Depressive, non-psychotic  298 - Psychosis, other  292 – Drug Psychosis  306 – Psychosomatic Disturbance/Sexual Dysfunction  787 – eating disorder | Q02, Q03, Q04,Q05, Q06, Q07,Q08, Q1, Q12, Q13, Q14, J13 |
| *Cardiac:*  427 – tachycardia, atrial flutter/fibrillation | E01, E05, E10, J09 |
| *Rheumatologic:*  733 – osteoporosis  795 – chronic fatigue  799 – malaise  796 – insomnia  726 – fibromyalgia  696 – psoriasis  714 – RA  710 – lupus  135 – sarcoid  555 – Crohn’s  564 – colitis | H04, H05, H10, J08, Q14, F06, H01, H07, H09, I01, I02, I08, I09 |
| *GI:*  564 – constipation  009 – diarrhea  579 - celiac | F12, F43, F82, F12 |
| *Other:*  785 – edema  788 – incontinence  257 – ED  704 – hair loss  Congenital anomalies (various)  277 – cystic fibrosis  290 – dementia/cognitive issues  315 – developmental delay  314 - ADHD | D01, O05, K04, K43, K06, I08, A01, A02, A03, A06, C01, L03, S02, J01, A84, Q01, Q09, Q10, Q81, Q82 |

1. **Any TSH done more frequently than every three months (+/- two weeks), excluding the following diagnoses in the past one year:**

| Condition | CIHI group |
| --- | --- |
| *Thyroid:*  193 – malignant neoplasm-thyroid  226 – benign neoplasm-thyroid  240 – simple thyroid goiter  242 – hyperthyroidism  244 - hypothyroidism  245 – thyroiditis  252 - parathyroid gland disorders | J03, J04, J05, J07, R10j |
| *Pregnancy:*  640 - threatened abortion, haemorrhage in early pregnancy  641 - abruptio placentae, placenta praevia  642- pre-eclampsia, eclampsia, toxaemia  643 - vomiting, hyperemesis gravidarum  644 - false labour, threatened labour  645 - prolonged pregnancy (post dates/post maturity pregnancy)  646 - other complications of pregnancy  651 - multiple pregnancy | M41, M42, M43, N41, N42, N43 |
| *Menstrual disorder:*  626 - amenorrhea, anovulation | L02, L03, L04, |
| *Nervous System:*  191 - malignant neoplasms – brain | R01A, R02C, R17, R18, J07 |
| *Psychiatric:*  296 – Bipolar Disorder  295 – Schizophrenia  300 – Depression/Anxiety  301 – OCD/Personality Disorder  311 – Depressive, non-psychotic  298 - Psychosis, other  292 – Drug Psychosis  306 – Psychosomatic Disturbance/Sexual Dysfunction  787 – eating disorder | Q02, Q03, Q04,Q05, Q06, Q07,Q08, Q1, Q12, Q13, Q14, J13 |
| *Cardiac:*  427 – tachycardia, atrial flutter/fibrillation | E01, E05 |

1. **Lipids** guideline: Canadian Cardiovascular Harmonized National Guidelines Endeavour (C-CHANGE) guideline for the prevention and management of cardiovascular disease in primary care: 2018 update. Tobe et al., 2018
2. **Any lipid testing in age < 40 years or if > 40 years and more often than every three years (+/- six months) excluding the following diagnoses**:

**the conditions noted overlap in ICD 9/10 diagnostic codes and the CIHI groups are even broader. Therefore, every condition considered may not be listed by name, but are encompassed in the appropriate CIHI group.*

| Condition | CIHI Group Associated |
| --- | --- |
| *Cardiac:*  401 – Hypertension  402 – Hypertensive heart  428 – Congestive Heart Failure  412 – Coronary Artery Disease  427 – Cardiac arrest  410 – MI  440, 443 – Vascular Disease  785 – Chest pain  441, 447 – Aneurysm  257 - ED | E01, E03, E04, E02, E05, E06, E07, E09, E10, E11, E12, E41, E43, E82, F07, K06 |
| *Respiratory:*  786 – dyspnea  496 – COPD  492 – Emphysema  277 - CF | D04, D03, J01 |
| *Nervous System:*  436 – stroke  350 - neuropathy | A41, A43, A84 |
| *Endocrine:*  278 – Obesity  272 – hyperlipidemia  796 – malnutrition  193 – malignant neoplasm-thyroid  226 – benign neoplasm-thyroid  240 – simple thyroid goiter  242 – hyperthyroidism  244 - hypothyroidism  245 – thyroiditis  626, 628 - PCOS  577 – pancreatitis, other disorders  250 – diabetes | J02, J10, J09, J13, J03, J04, J05, J08, G03, L01 |
| *Psychiatry:*  303 – alcohol abuse  305- smoking  296 – Bipolar Disorder  295 – Schizophrenia  300 – Depression/Anxiety  301 – OCD/Personality Disorder  311 – Depressive, non-psychotic  298 - Psychosis, other  292 – Drug Psychosis  306 – Psychosomatic Disturbance/Sexual Dysfunction | Q02, Q04, Q05, Q08, Q07 |
| *Renal:*  580 – nephropathy  585 – chronic renal failure | K41, K01 |
| *Eye:*  379 – retinopathy, xanthalasma | B02, I09 |
| *Rheumatologic:*  710 – lupus  696 – psoriasis  714 – RA  555 – Crohn’s disease  564 – colitis  274 – gout | F06, H01, H03,H07, H09, I01, I02 |
| *GI:*  574, 156 – gallbladder disease  070 – hepatitis, fatty liver, cirrhosis | F01, F81, G05 |
| *Other:*  044 – HIV  758 – Down’s Syndrome | P01, S02 |

1. **Any lipid screening more frequently than every three months +/- two weeks excluding the following diagnoses** (circumstances may include uncontrolled disease and/or medication adjustments):

| Condition | CIHI group |
| --- | --- |
| *Cardiac:*  401 – Hypertension  402 – Hypertensive heart  428 – Congestive Heart Failure  412 – Coronary Artery Disease  427 – Cardiac arrest  410 – MI  440, 443 – Vascular Disease  785 – Chest pain  441, 447 – Aneurysm | E01, E03, E04, E06, E09, E11, E12, E41, E43, F07 |
| *Nervous System*:  436 - stroke | A41, A43, A84 |
| *Endocrine:*  278 – Obesity  272 – hyperlipidemia  796 – malnutrition  193 – malignant neoplasm-thyroid  226 – benign neoplasm-thyroid  240 – simple thyroid goiter  242 – hyperthyroidism  245 – thyroiditis  250 - diabetes | J09, J10, J13, J03, J04, J05, J08, J02 |
| *Psychiatry:*  303 – alcohol abuse  305 – smoking | Q07 |
| *Renal:*  580 – nephropathy  585 – chronic renal failure | K41, K01 |
| *Rheumatologic:*  710 – lupus  696 – psoriasis  714 – RA  555 – Crohn’s disease  564 – colitis  274 – gout | F06, H01, H07, H09, I01, I02 |
| *Skin:*  706 - acne | I04 |
